# *Chlamydia trachomatis* inhibits NF-κB-dependent ferroptosis through PARP10 upregulation to promote reproduction

**DOI:** 10.1128/spectrum.03568-25

**Published:** 2026-05-21

**Authors:** Xinglv Wang, Yewei Yang, Hongrong Wu, Yu Zhou, Jinrong Zhang, Lili Chen, Zhongyu Li

**Affiliations:** 1 Institute of Pathogenic Biology, School of Nursing, Hengyang Medical School, University of South China 159373, Hengyang, Hunan, China; University of Louisville, Louisville, Kentucky, USA

**Keywords:** *Chlamydia trachomatis*, ferroptosis, cell death, PARP10, NF-κB pathway

## Abstract

*Chlamydia trachomatis* (*Ct*)*,* an obligate intracellular pathogen, manipulates host cell death pathways to establish infection. Although ferroptosis—an iron-dependent cell death process marked by lipid peroxidation—constitutes an antimicrobial defense mechanism, its regulation during *Ct* infection remains in the early stages of exploration. Here, we report that *Ct* actively suppresses ferroptosis in host cells, with maximal inhibition at 24 hours post-infection. This suppression was demonstrated through attenuated malondialdehyde production, reduced Fe²^+^ accumulation, and preserved mitochondrial integrity. Transcriptomic analysis of *Ct*-infected cells identified PARP10 as a prominently upregulated host factor. Crucially, we discovered that *Ct*-induced PARP10 inhibits NF-κB activation, leading to downregulation of ferroptosis-promoting genes. Disruption of the PARP10/NF-κB axis restored ferroptosis and impaired *Ct* replication. Our findings unveil a non-canonical strategy wherein *Ct* exploits PARP10 to repress NF-κB-dependent ferroptotic defense, thereby maintaining iron/lipid peroxide homeostasis for intracellular survival. Targeting this pathway may offer novel anti-chlamydial therapeutics.

**IMPORTANCE:**

*Chlamydia trachomatis* is a widespread bacterial infection and a leading cause of preventable blindness and sexually transmitted diseases. A key to its success is its ability to survive inside our cells by disarming the body's innate defense systems. We discovered that the bacteria actively block a potent form of cell death called ferroptosis by exploiting a human protein, PARP10. This previously unknown strategy allows *Chlamydia* to create a safe haven for its replication. Uncovering this novel bacterial survival mechanism not only deepens our understanding of the infection process but also reveals the PARP10 pathway as a promising new target for developing much-needed therapeutic drugs against this pervasive pathogen.

## INTRODUCTION

*Chlamydia trachomatis* (*Ct*) infection is an important public health problem worldwide, causing mainly trachoma and sexually transmitted diseases. According to the World Health Organization (WHO) 2021 report, there are approximately 127 million new cases of genital tract *Ct* infections globally each year, making it the most common bacterial sexually transmitted infections (STIs). As *Ct* infections tend to be insidious, they lead to a prolonged course and serious complications, including pelvic inflammatory disease, ectopic pregnancy, and infertility in women, as well as epididymitis and potential infertility in men (1, 2). Therefore, it is particularly important to study the pathogenic mechanisms and control of *Ct* (3).

*Ct* is a strictly intracellular parasitic pathogen. Two different morphological structures can be observed under the electron microscope: one is small and dense particles called elementary bodies (EBs; diameter of about 0.2 μm), which are infectious. The other is large, loose reticulum bodies (RBs; diameter of about 1 μm), which are uninfectious. The unique developmental cycle of *Ct* from EBs-RBs-EBs is completed within the inclusions. To accomplish intracellular proliferation, *Ct* must be dependent on the host for nutrients (4) and ensures the completion of growth and development by reprogramming host cell metabolism, modulating signaling pathways, and manipulating cell death (5). Previous studies have shown that *Ct* regulation of apoptosis has a time effect, that is, in the early stage of infection, *Ct* inhibits host cell apoptosis and promotes reproduction by regulating the Bcl-2 family, PI3K/Akt pathway, and so forth (6, 7). In the late stage of infection, *Ct* induces apoptosis and promotes its release for propagation (8). The regulation of autophagy is also dual: on the one hand, it inhibits host defensive autophagy, and on the other hand, it may use autophagy-related structures to promote infection (9).

Ferroptosis is a novel mode of programmed cell death that differs from other forms such as apoptosis and necrosis and is characterized by the iron dependent accumulation of lipid peroxidation to lethal levels. The occurrence of ferroptosis is mainly dependent on increased accumulation of metabolites reactive oxygen species (ROS), phospholipids containing polyunsaturated fatty acid chains (PUFA-PL), and iron. Therefore, mechanistic studies of ferroptosis have focused on three classical pathways: imbalance of amino acid antioxidant system, disruption of iron metabolism, and lipid peroxide accumulation (10). Iron is an essential nutrient for both host cells and pathogens, and iron regulation can influence the outcome of infection. On the one hand, the host limits the ability of pathogens to acquire iron by activating a series of defense mechanisms. For example, the host sequesters iron through iron-binding proteins (e.g., transferrin and ferritin), which reduces iron acquisition by pathogens (11). Following infection, host tissues alter local iron homeostasis through enhanced iron uptake and macrophage sequestration of iron, heme, and so forth (12). On the other hand, pathogens have evolved multiple iron acquisition mechanisms to resist host defenses (13). Ferroptosis sensitivity is regulated by import, storage, and export of iron (14, 15). To date, the only putative iron acquisition system identified in *Ct* is the ATP-binding cassette (ABC) permease YtgABCD, and YtgA is the metal-recruiting component of YtgABCD and is likely involved in the acquisition of ferrous iron from host cells (16). Thus, the ability of *Ct* to take up iron and maintain iron homeostasis may be critical for the regulation of host ferroptosis.

Studies have shown that intracellular pathogens manipulate ferroptosis to enhance their survival and proliferation within the host in several ways (17, 18). For example, EBV inhibits ferroptosis by promoting the transcription factor NRF2 upstream of GPX4 to promote tumorigenesis. Or, it induces ferroptosis and promotes pathogen replication or dissemination (e.g., Mycobacterium tuberculosis [19], and Cryptococcus neoformans [20], etc.) by inhibiting GSH or GPX4 expression. Recent reports indicate that during late infection (48–72 hpi), *Ct* induces ferroptosis via SLC7A11/GPX4, thereby promoting replication and progeny release (21, 22). This finding offers a novel explanation for the cellular lysis process observed in the terminal phase of infection. However, given *Ct*’s unique developmental cycle, host cell survival must be maintained during its critical stages of infection establishment, replication, and amplification (early and mid-infection). Does *Ct* actively suppress ferroptosis to prevent premature clearance by host defenses before completing replication? Currently, there is a lack of in-depth research on how *Ct* regulates ferroptosis during early infection to maintain its intracellular survival niche. Therefore, my work provides a novel perspective on the temporal regulation of host cell death by intracellular bacteria and identifies ferroptosis suppression as a critical strategy for *Ct* during its establishment phase.

In this study, human cervical cancer epithelial cells were used as model cells to construct an *in vitro* model of E-type *Ct* acute infection. Cell viability and lipid peroxidation production were first evaluated at different times after ferroptosis stress and *Ct* infection of HeLa cells. Then, changes in ferrous ions and mitochondria were observed by fluorescence microscopy and electron microscopy. The role of PARP10 in *Ct* infection and ferroptosis was further determined mechanistically. This study reveals a new mechanism by which *Ct* resists host cell death and promotes its own proliferation, providing an important basis for exploring the pathogenic mechanism and potential therapeutic targets of *Ct*.

## MATERIALS AND METHODS

### *Chlamydia trachomatis* and cell culture

The human cervical epithelial cell line HeLa 229 (ATCC CCL-2.1) was selected for its established susceptibility to *Ct* infection. The cells were cultured and maintained in DMEM (Dulbecco’s modified Eagle’s medium) containing 10% fetal bovine serum (FBS; Gibco) at 37°C in a 5% CO₂ incubator. For infection, subconfluent monolayers were pretreated with serum-free medium containing DEAE-dextran (30 μg/mL) for 10 min at 37°C to enhance bacterial adherence. *Ct* serovar E strain (ATCC VR-348B) was propagated and titrated to determine inclusion-forming units (IFU). *Ct* was diluted in serum-free medium to achieve a multiplicity of infection (MOI) of 2 based on preliminary optimization assays. Inoculum was added to cells and centrifuged at 1,200 rpm for 30 min at 37°C to synchronize infection. After centrifugation, the inoculum was aspirated, and fresh DMEM supplemented with 10% FBS was added. The ferroptosis inducer Erastin (from a DMSO stock solution) or an equivalent volume of vehicle control (DMSO) was added immediately at this time point (0 hour post-infection, hpi). Based on the developmental cycle of *Ct*, infected and treated cells were incubated at 12 hpi, 24 hpi, and 40 hpi (mid-to-late stages after inclusion formation), then harvested for subsequent analysis.

To prepare clarified chlamydial lysates for reinfection studies, infected HeLa cells were washed 3–5 times with PBS and scraped into sucrose-phosphate-glutamate (SPG) buffer. The suspension was then sonicated on ice (20% power) and centrifuged at 3,000 rpm for 15 min at 4°C. The resulting supernatant was used immediately for the next round of infection.

### Cell viability assay

Cell viability was assayed using the maximum sensitivity cell counting kit-8 (CCK-8) according to the manufacturer’s instructions (#KTA2030, Abbkine, #KTA2030, China). Cultured HeLa cells were inoculated in 96-well plates at a density of 5,000 cells/well, with five replicate wells in each group, and allowed to adhere to the wall overnight. Culture medium containing 10% CCK-8 was added to each well and incubated away from light for 30 min–1 h. Absorbance at 450 nm was detected by a microplate reader.

### Determination of malondialdehyde (MDA) and lipid peroxidation

MDA is a natural product of lipid oxidation in organisms and is widely used as an indicator of lipid oxidation. Cells spread in six-well plates were lysed on ice, and thiobarbituric acid included in the kit was added to the supernatant of the cell homogenate to form a TBA-MDA mixture according to the manufacturer’s instructions (Beyotime, China), followed by colorimetric assay at 532 nm.

BODIPY 581/591 C11 (Thermo Fisher, USA) can be used to detect reactive oxygen species (ROS) in cells and cell membranes. Referring to the instruction manual, 198 μL of DMSO was added to the reagent containing 1 mg of BODIPY 581/591 C11 and blown up with a pipettor to obtain 10 mM of BODIPY 581/591 C11 master mix, and an appropriate amount of 10 μM of BODIPY 581/591 C11 working solution was prepared by diluting it with serum-free medium. In this study, we chose to use the 488 nm excitation of the flow cytometer to detect FL1 (Excitation light at 530 nm), in which only the oxidized state was detected. All the above assays were performed in three independent replicate experiments.

### Fe^2+^ detection

HeLa cells were seeded in 24-well plates and cultured overnight at 37°C in a 5% CO₂ incubator. HeLa cells were infected with *Ct* and added with appropriate inducers, and cultured in the incubator for 24 h. According to the instructions, add 35 μL of DMSO into a tube containing 24 μg of FerroOrange (Dojindo), avoid light, and blow with a pipettor to obtain a 1 mM FerroOrange solution, and then dilute it with serum-free medium to prepare a 1 μM FerroOrange working solution (the working solution is unstable, and should be prepared and used now). Remove the 24-well plate from the incubator, aspirate the original medium from the 24-well plate, wash three times with serum-free medium, and then incubate the plate with Hoechst 33342 and FerroOrange working solution successively at 37°C in a 5% CO₂ incubator. The incubation was carried out under a fluorescence microscope or fluorescence microplate reader directly without washing.

### Mitochondrial membrane potential (MMP) analysis

Fluorescence microscopy was performed to observe the MMP of HeLa cells stained with JC-1 (C2006, Beyotime). Carbonyl cyanide 3-chlorophenylhydrazone (CCCP) was used as a positive control to treat the cells in advance for 20 min. 1 mL of cell culture medium and 1 mL of JC-1 staining solution were added to a six-well plate, which was incubated at 37°C in a cell culture incubator for 30 min. After incubation, the cells were washed three times with JC-1 staining buffer. Cell culture solution was added and observed under a fluorescence microscope. The red/green fluorescence intensity ratio was used to assess MMP.

### Transmission electron microscopy (TEM) analysis

The culture medium was discarded, trypsin was added to digest the cells for 3 min, and the cells were collected in 1.5 mL centrifuge tubes. The cells were centrifuged at 1,200 rpm for 5 min, the supernatant was discarded, and the cells were fixed by adding 2.5% glutaraldehyde at 4°C for 12 hpi. The samples were sent to the Shiyanjia Lab (www.shiyanjia.com) for subsequent work.

### Immunofluorescence analysis (IFA)

The cells were fixed with 4% paraformaldehyde for 30 min, washed three times with PBS, and then 0.3% TritonX-100 was added, and the cells were permeabilized for 10 min at room temperature. The cells were washed three times with PBS and then closed incubated in a DMEM incubator containing 10% FBS for 1 h. After washing twice with PBS, the cells were incubated for 1 h in the incubator with DMEM diluted with primary antibodies (rabbit anti-*Ct*, 1:500; mouse anti-p65, 1:50, ZenBio) at 4°C overnight. The following day, secondary anti-rabbit (Dylight 488, 1:200; BA1146, Boster) and anti-mouse (Dylight Cy3, BA1031, Boster) fluorescent antibodies, diluted in DMEM protected from light, were incubated at 37°C for 1 h. Finally, nuclei were stained using DAPI and photographed by fluorescence microscopy.

### Quantitative Real-Time PCR (qRT-PCR)

Total RNA was extracted from cells with Trizol reagent and then reverse transcribed to cDNA using a reverse transcription kit (Tiangen, China) according to the instructions. qRT-PCR was performed in a LightCycler 96 instrument (Roche, Basel, Switzerland) using SYBR Green premix (Tiangen). PCR was performed in a LightCycler 96 instrument (Roche, Basel, Switzerland) under real-time PCR conditions of 600 s of amplification at 95°C, followed by 40 cycles of 95°C for 15 s and 60°C for 30 s. 18S rRNA was used as an internal control. The primers were as follows: PARP10: 5′-TGGTGGAGATGGTGCTATTGATGG-3′ (forward primer) and 5′-CCGCAAGAAGGTCCTCATGGTAG-3′ (reverse primer); 18S rRNA: 5′-CGCTCGCTCCTCTCCTACTT-3′ (forward primer) and 5′-CGGGTTGGTTTTGATCTGATAA-3′ (reverse primer). All reactions were performed in triplicate. The relative amounts of mRNA were calculated by using the comparative 2(−ΔΔCt) method.

### RNA interference

The three siRNAs used to knock down PARP10 were purchased from RiboBio (Guangzhou, China). The genOFFTM st-h-PARP10_001 target sequence is GGTAGAGGGATTATGACAA. The genOFFTM st-h-PARP10_002 target sequence is CTACCATGAGGACCTTCTT. The genOFFTM st-h-PARP10_003 target sequence is GCAGCATTAGCTGCCATGT. According to the transfection instructions, when the cell density reached about 50%, the adherent cells were transfected with Lipofectamine 3000 (Invitrogen).

### Western blot analysis

Whole cells were lysed in RIPA high-performance lysate containing protease and phosphatase inhibitors. The concentration of protein samples was determined by the BCA method (Beyotime, Shanghai, China). Then, 20 μg of the sample was separated by 10% SDS/PAGE and transferred to PVDF (0.22 mm; Millipore, Bedford, MA, USA). After 1 h of closure at room temperature in 5% skimmed milk, the samples were incubated with anti-PARP10 (1:1,000, abs143444; Absin, China), Phospho-NF-κB p65 (1:1,000, TA2006; Abmart, China), NF-κB p65 (1:2,000, T55034; Abmart, China), Phospho-IκBα (1:1,000, #2859; Cell Signaling Technology, U.S.A.), IκBα (1:1,000, #9242; Cell Signaling Technology, U.S.A.), β-actin (1:4,000, R380624, zZen-bBio) overnight at 4°C. Secondary antibody (goat anti-rabbit, 1:6,000, RS0002, Immunoway, China) was incubated at 37°C for 1 h. Finally, the results were visualized using an ultrasensitive ECL luminescent solution (BMU102, Abbkine, China) and enhanced Chemiluminescent Western Blot Imaging System G: BoxChemi XXX9 (Syngene, Cambridge, UK).

### Statistical analysis

Statistical analysis and visualization were performed using GraphPad Prism (version 10.1.2). All experimental data are presented as mean ± standard error. Prior to parametric testing, data normality and homogeneity of variance were assessed. Normality was evaluated using the Shapiro-Wilk test; homogeneity of variance was assessed using the Brown-Forsythe test (or Bartlett’s test). For data meeting assumptions of normality and homogeneity of variance, Student’s *t*-test was used for two-group comparisons, and one-way analysis of variance (ANOVA) for multiple-group comparisons. For data failing normality or homogeneity of variance assumptions, corresponding nonparametric tests were applied: Mann-Whitney *U*-test for two-group comparisons and Kruskal-Wallis test for multiple-group comparisons. *P* < 0.05 was considered statistically significant.

## RESULTS

### *Ct* has a significant time effect on the regulation of ferroptosis

**Fig 1:**
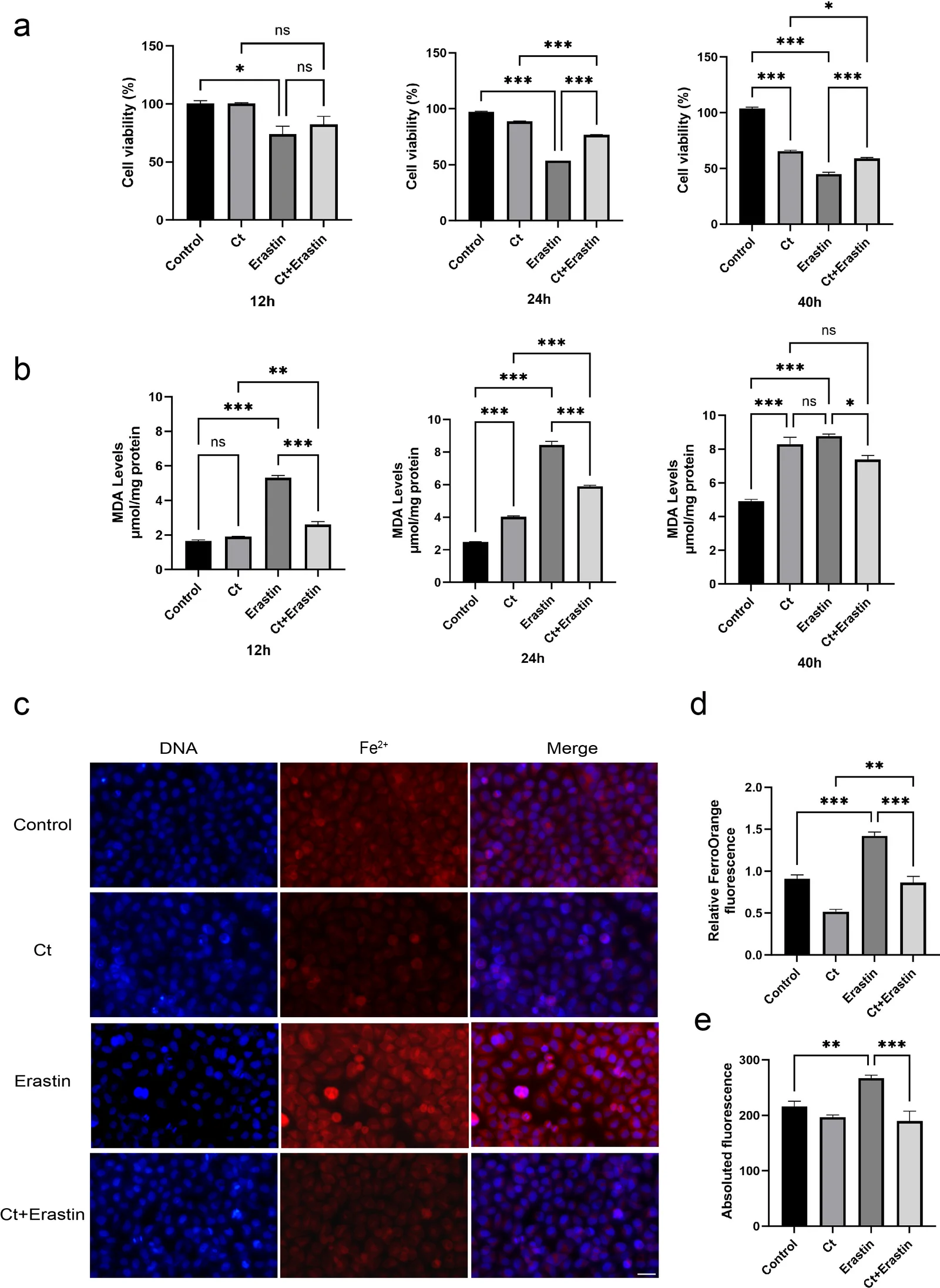
Phenotypic studies of *Ct* inhibition of ferroptosis. (**a**) CCK-8 assay for cell viability (Erastin treatment time synchronized with infection). (**b**) MDA concentration in cell lysates according to kit manufacturer’s instructions. (**c**) FerroOrange assay for intracellular Fe²^+^ (400 ×). DNA (blue) stained with Hoechst 33,342. Data were normalized to the mean fluorescence intensity of the uninfected control group. (**d**) Relative fluorescence intensity of FerroOrange. (**e**) Absolute fluorescence intensity of FerroOrange in fluorescence microplate reader. *P < 0.05; **P < 0.01; and ***P < 0.001; ns, no significance. All data are expressed as mean ± SEM (*n* = 3) and statistically analyzed by analysis of variance. The control group was added with DMSO to exclude solvent interference.

Previously, the regulation of apoptosis by *Ct* had a significant time effect, inhibiting and inducing apoptosis in host cells at early and late stages of infection, respectively (8, 23). Therefore, we speculated that the regulation of ferroptosis by *Ct* is similar. Empirically, three typical time points (12 hpi, 24 hpi, and 40 hpi) during the RBs to EBs transition period in the *Ct* developmental cycle were selected (24), and the ferroptosis inducer Erastin (25) was used. HeLa cells were stimulated with a concentration of Erastin of 40 μM (26), and cell viability was determined by CCK-8 (Fig. 1a). It was found that Erastin played an effective role in all three time points. Compared with the control group, *Ct* started to show a decreasing trend in cell viability at 24 hpi, with a significant decrease at 40 hpi. Surprisingly, however, *Ct* showed the ability to rescue the decrease in cell viability caused by Erastin at all time points, with a marked effect starting at 24 hpi. In addition, cell viability was reduced in the infected group with the addition of Erastin inducer compared to single *Ct* stimulation. Therefore, we speculate that there is a mutual resistance between *Ct* and ferroptosis. In other words, *Ct* infection suppresses the ferroptosis process in host cells, while the induction of ferroptosis in turn impairs *Ct* proliferation.

Next, to probe the level of oxidative stress, we determined the production of malondialdehyde (MDA), a metabolic end product of lipid peroxidation (Fig. 1b). A significant elevation of MDA was observed in Erastin-treated HeLa cells. At the same time, we observed that at 40 hpi of infection, the MDA produced by the *Ct* group was elevated to a level comparable to the Erastin group, which may be attributed to the fact that infection peaked at 40 hpi, when the EBs were killing or had already killed the hosts and ruptured the membranes. Whether part of the elevated MDA was produced by *Ct* itself is unknown. On the one hand, when we focused on the Erastin group and the *Ct* + Erastin group, we found that *Ct* exhibited resistance to ferroptosis induced by Erastin regardless of the infection time point. On the other hand, when we focused on the *Ct* group and the *Ct* + Erastin group, it stood to reason that under the double burden of *Ct* and Erastin on HeLa, there would be lower cell viability as well as more MDA production than that stimulated by infection alone. However, up to 40 hpi of infection, the *Ct* + Erastin group appeared to produce less MDA than the *Ct* group, which to some extent responded to our earlier speculation that *Ct* was resisting ferroptosis while ferroptosis was inhibiting *Ct* proliferation. Finally, we selected 24 hpi of infection as the time point for subsequent studies.

### *Ct* prevents Erastin-induced iron overload and mitochondrial damage

We have observed changes in the oxidative stress marker MDA, and iron overload is usually associated with oxidative stress. And the initiation of ferroptosis depends on the availability of ferrous iron (Fe²^+^) (27). Iron is an essential nutrient for the growth and proliferation of *Ct* (28). So how does *Ct* cope with excess intracellular iron? We next used FerroOrange, which enables simple and rapid fluorescence imaging of Fe^2+^ within living cells. Erastin treatment significantly increased Fe^2+^ levels, not through directly introducing iron but by disrupting cellular iron homeostasis—specifically by inhibiting System Xc-− and its downstream effects—thereby indirectly increasing the unstable pool of catalytically active iron (29). However, we observed here that *Ct* ingeniously balanced or prevented excessive intracellular Fe^2+^ accumulation (Fig. 1c through e). It has been reported that the only putative iron acquisition system found in *Ct* is the ATP-binding cassette (ABC) permease YtgABCD, where YtgA is the metal-recruiting component of YtgABCD and is likely involved in the acquisition of ferrous iron from host cells (16).

**Fig 2:**
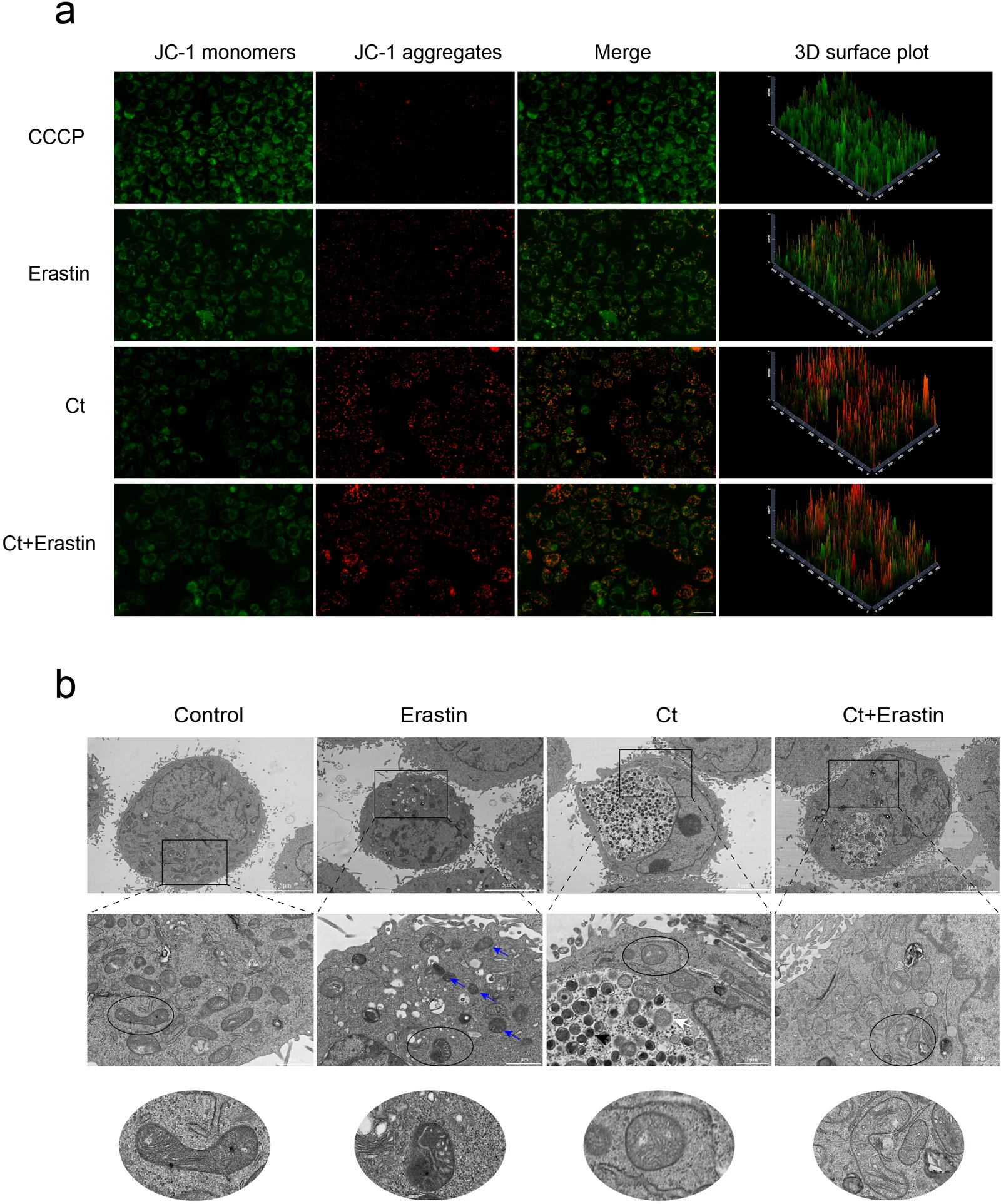
*Ct* attenuates Erastin-induced mitochondrial damage. (**a**) Fluorescence microscopy (400 ×) was performed to observe the fluorescence intensity of JC-1 in mitochondria, and the positive control was treated with 10 μM CCCP for 20 min prior to the assay. (**b**) Transmission electron microscopy was performed to observe inclusions and mitochondria at magnifications of 3,000 ×, 10,000 ×, and Zoom, respectively, from top to bottom. Blue arrowheads indicate the typical mitochondrial morphology of ferroptosis, black arrowheads indicate EBs, and white arrowheads indicate RBs.

The central site of iron metabolism is the mitochondria, and mitochondrial dysfunction leads to oxidative stress and cellular damage (30). Indeed, *Chlamydia* can disrupt apoptosis by remodeling host cell mitochondria and thereby disrupting cell death (31). Therefore, we observed the morphology of inclusions and mitochondria by transmission electron microscopy as well as the mitochondrial membrane potential (MMP) using the JC-1 probe. In most cells, MMP was reduced after CCCP treatment, and JC-1 staining showed green fluorescence (monomers), whereas normal cells should show red fluorescence (aggregates). As shown in Fig. 2, Erastin-treated HeLa cells showed mitochondrial atrophy, increased density, decreased cristae, and rupture of the outer membrane; MMP decreased (Fig. S1a). These are the hallmarks of mitochondrial damage in ferroptosis. However, such changes were rarely observed in *Ct*-concomitantly treated cells. Instead, *Ct* rescued the adverse effects of Erastin, including iron overload and mitochondrial damage.

### Ferroptosis affects the proliferation of *Ct*

**Fig 3:**
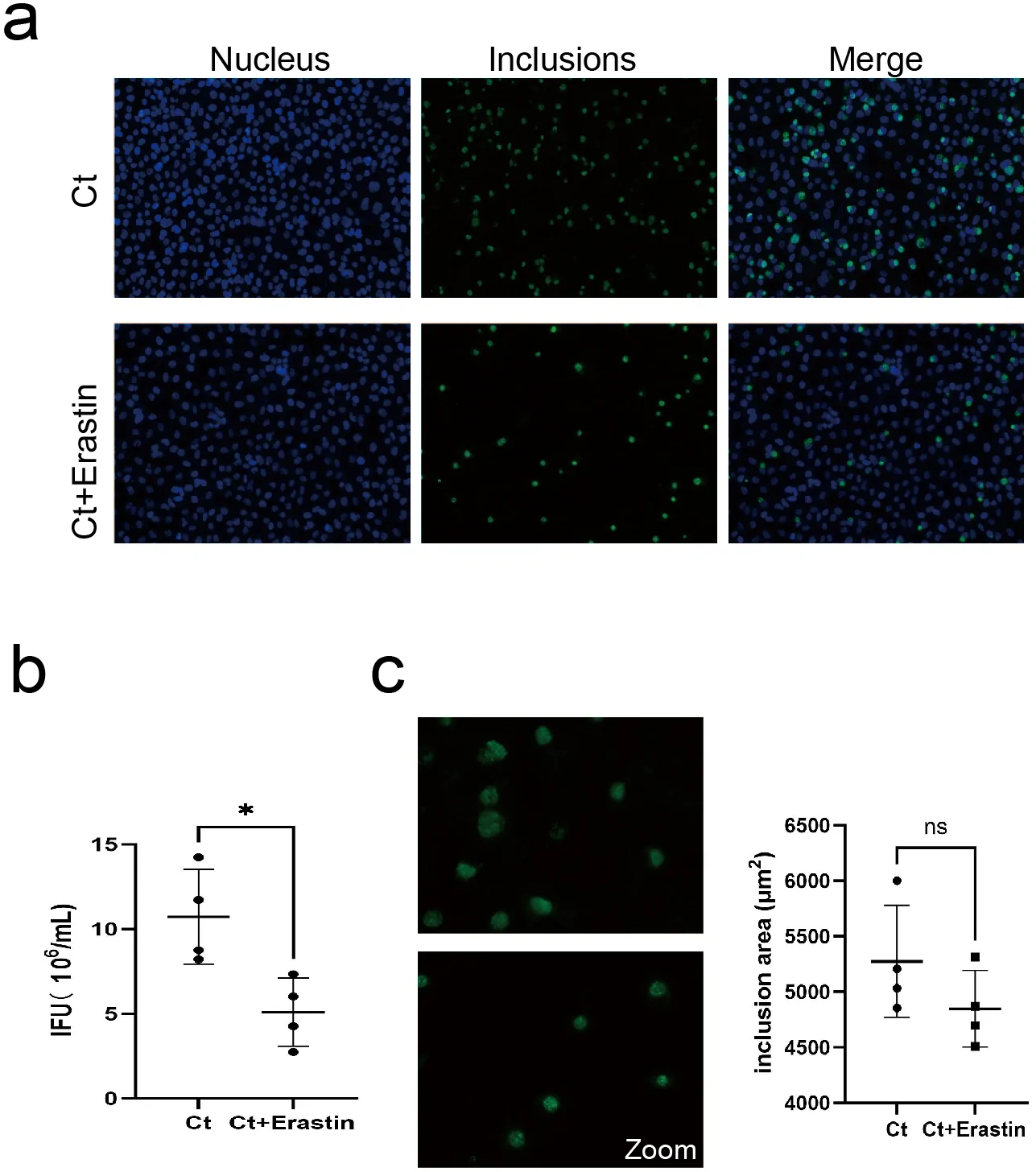
Ferroptosis inhibits *Ct* proliferation. (**a**) Fluorescence microscopy (200 ×) of *Ct* progeny inclusions during the reinfection assay. DNA was stained with DAPI (blue), and inclusions were stained with Dylight 488 (green). IFUs (**b**) and inclusions sizes (**c**) were calculated by the ImageJ software. Data are expressed as mean ± SEM (*n* = 5) and analyzed by Student’s *t*-test. *P < 0.05; ns, not significant (*P* ≥ 0.05).

Results in Fig. 2b indicate that bacterial maturation and proliferation are impaired under ferroptosis stress. To assess the functional impact of this defect on bacterial progeny, we collected chlamydial lysates from the *Ct* and *Ct* + Erastin groups approximately 40 hpi and used them to reinfect fresh HeLa cells in the absence of Erastin (Fig. 3a). When IFUs were measured 24 hours after reinfection, the infectivity of progeny derived from the *Ct* + Erastin group was significantly reduced compared to the *Ct* group (Fig. 3b). This indicates that Erastin-induced ferroptosis stress during the initial infection impairs the infectivity of subsequent bacterial generations. Notably, however, the inclusions formed by these progeny during reinfection appeared normal in size, showing no significant difference compared to those formed by progeny from the *Ct*-only group (Fig. 3a and c). This indicates that the inhibition of inclusion development observed during the initial Erastin treatment (Fig. 2b) is reversible after the resolution of ferroptosis stress.

### *Ct* inhibits ferroptosis by upregulating PARP10

**Fig 4:**
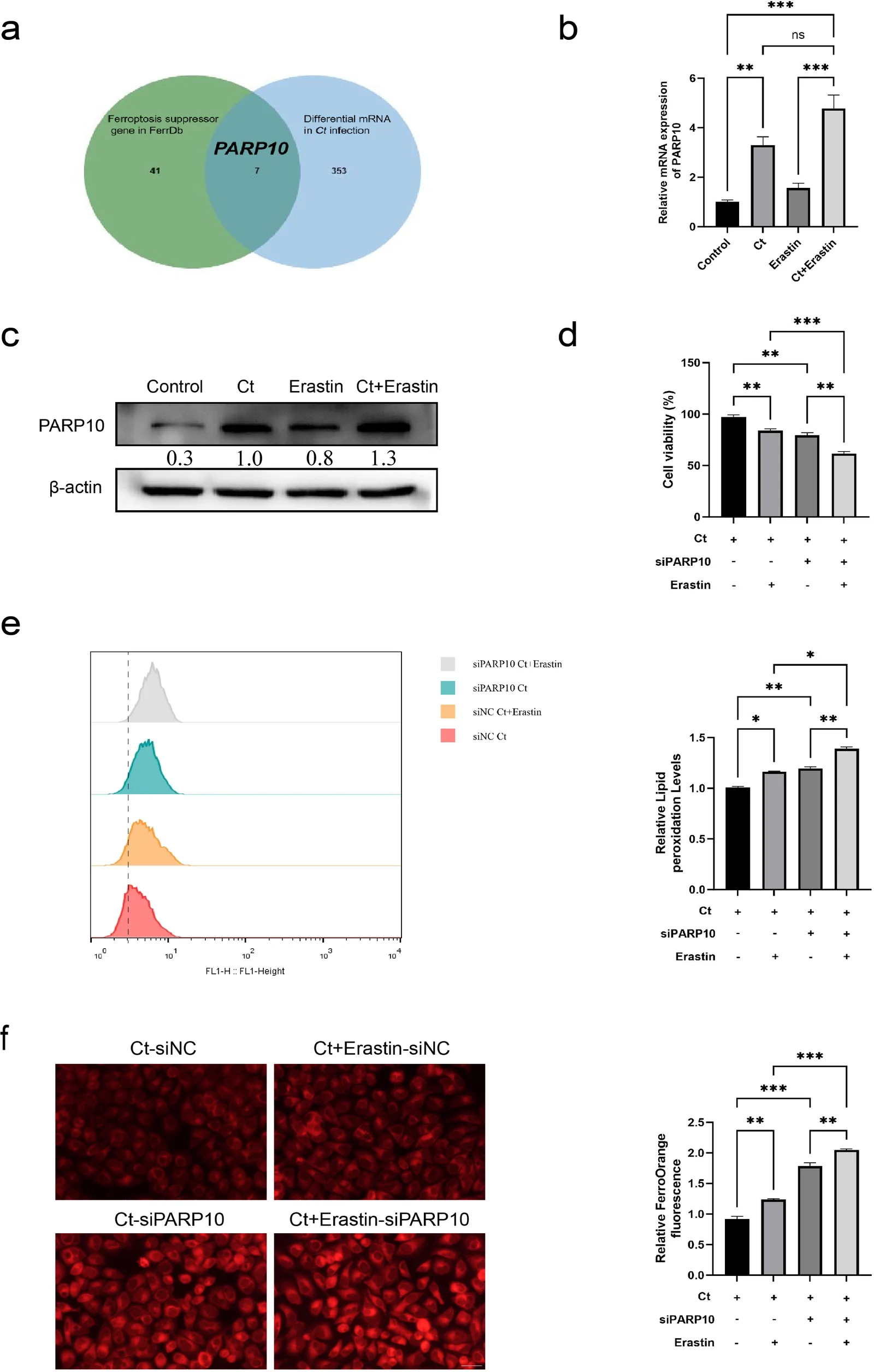
*Ct* suppresses ferroptosis by up-regulating PARP10. (**a**) Venn diagram showing the transcriptional profile of *Ct* acute infection and the intersection of the ferroptosis suppressor gene in FerrDb selected for PARP10. (**b**) qRT-PCR to verify PARP10 mRNA levels. (**c**) Western blot to detect PARP10 protein levels. The Western blot image is representative of experiments showing consistent results. Numbers below the bands indicate the relative density normalized to β-actin, derived from this blot. (**d**) HeLa cells transfected with siPARP10 were treated with *Ct* and/or Erastin for 24 hpi, CCK-8 was used to detect cell viability, flow cytometry was used to detect ROS in the cells and cell membranes (**e**), and Fe^2+^ was visualized by FerroOrange (400 ×) (**f**). Fluorescence intensity quantification was read by ImageJ. All data were expressed as mean ± SEM (*n* = 3) and statistically analyzed by ANOVA. *P < 0.05; **P < 0.01; ***P < 0.001; ns, not significant (*P* ≥ 0.05).

Based on the phenotype, we next explored the underlying molecular mechanisms. Gene expression profiles of *Ct* acute infection previously constructed were screened with diffExp.gene.log2 (fc) ≥1 or ≤ −1, *P* ≤ 0.05. The 360 differential genes screened were taken as intersections with ferroptosis suppressor genes (http://www.zhounan.org/ferrdb). We finally selected the gene to be studied next-PARP10 from the intersection set (Fig. 4a). PARP10 (Poly (ADP-Ribose) Polymerase Family Member 10) is a protein-coding gene. PARP inhibitors are able to induce ferroptosis in tumor cells and are important targets for cancer therapy (32). First, to verify that *Ct* up-regulates host PARP10, we examined the transcript level and protein level by qPCR and Western blot, respectively (Fig. 4b and c). The results showed that the expression of PARP10 in the *Ct* group was significantly higher than the control group, which was consistent with our transcriptional profiling data. More importantly, the high expression of PARP10 in cells treated with both *Ct* and Erastin revealed that *Ct* might be inhibiting ferroptosis by upregulating PARP10. Next, we knocked down PARP10 and assayed ferroptosis-related phenotypes (cell viability, intracellular ferrous iron, and lipid peroxidation). ROS generated by apoptotic pathways localize to the mitochondrial intermembrane space and cytoplasm. In contrast, ROS produced during ferroptosis primarily originate from cell membranes (particularly the plasma membrane and organelle membranes) (33). Therefore, we opted for the C11-BODIPY(581/591) fluorescent probe (34) instead of DCFH-DA (which detects total ROS). This probe primarily reacts with free radicals within the cell membrane, and hydroxyl radicals possess exceptionally strong oxidative capacity. The results showed that ferroptosis was more obvious in cells infected with *Ct* with low expression of PARP10. It not only decreases cell survival (Fig. 4d) but also increases lipid peroxidation levels (Fig. 4e) and intracellular ferrous (Fig. 4f), which indicates that PARP10 and ferroptosis are negatively correlated. Moreover, this restoration of ferroptosis via PARP10 knockdown significantly reduced the yield of infectious progeny, as determined by an IFU assay (Fig. S1b). Altogether, these results elucidate the survival strategy of *Ct* to resist host ferroptosis by upregulating PARP10.

### PARP10 inhibits ferroptosis by suppressing NF-κB signaling pathway

**Fig 5:**
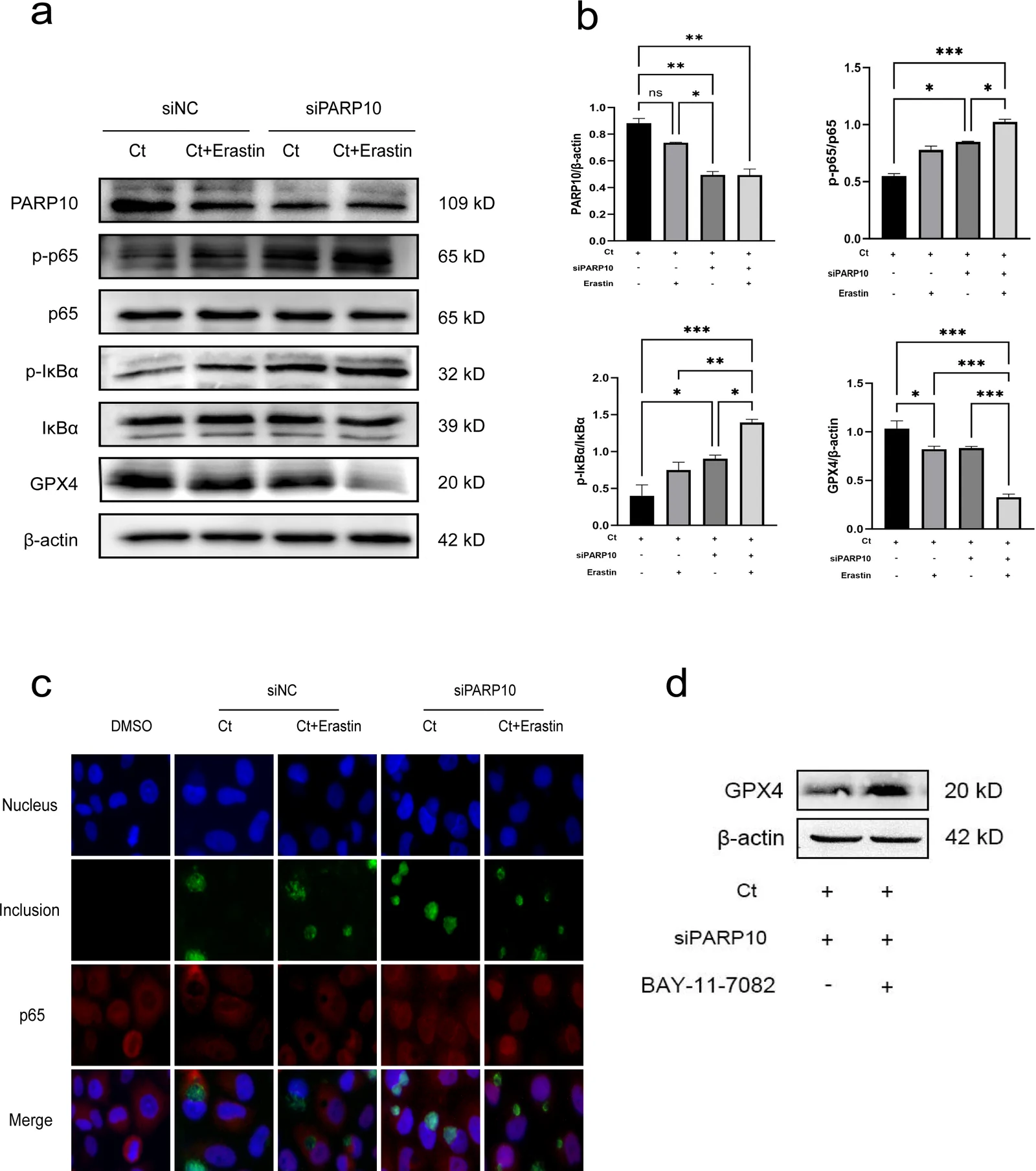
PARP10 suppresses ferroptosis by inhibiting the NF-κB signaling pathway. (**a**) HeLa cells with PARP10 knockdown were treated with *Ct* and/or erastin for 24 hpi, followed by whole-cell lysis collection. Western blot was performed to analyze the phosphorylation levels of p65 (Ser536) and IκBα (Ser32), as well as the total protein levels of p65, IκBα, and GPX4. β-actin served as the internal loading control. (**b**) Grayscale intensity quantification was conducted using ImageJ. Data are presented as mean ± SEM (*n* = 3). (**c**) Indirect immunofluorescence was used to observe p65 nuclear translocation across groups. Cells were fixed with formaldehyde and permeabilized with 0.3% Triton X-100. Nuclei (blue), inclusions (green), and p65 (red) were stained with DAPI, DyLight 488, and DyLight Cy3, respectively. p65 subcellular localization was analyzed by immunofluorescence microscopy (1,000 × magnification). (**d**) HeLa cells were pretreated with the NF-κB inhibitor (BAY 11-7082) at a concentration of 30 μM for 30 minutes. GPX4 expression was analyzed by Western blot at 24 hours post-infection (hpi). *P < 0.05; **P < 0.01; ***P < 0.001; ns, not significant (*P* ≥ 0.05).

PARP10 contains negative regulation of NF-κB transcription factor activity and negative regulation of K63-linked ubiquitinated proteins in significantly enriched GO_Term. PARP10 has been reported to interfere with the polyubiquitination of NEMO by interacting with K63-pUb (35), while NEMO and IKKα/IKKβ together form the IκB kinase (IKK) complex. In the resting state, IκBα, β, and ε bind to the NF-κB dimer (p50/p65), preventing its nuclear translocation and thus inhibiting activation of the NF-κB signaling pathway. In addition, a previous study showed that *Ct* ChlaDub1 inhibits the ubiquitination degradation of IκBα and stabilizes the IκBα/p50/p65 complex (36), as well as that *Chlamydia* protease-like activator CPAF inhibits the phosphorylation of IκBα and thus cuts off the nuclear translocation of p50/p65 (37). There is now much evidence linking the NF-κB signaling pathway to ferroptosis (38). In Erastin-induced ferroptosis in smooth muscle cells, the phosphorylation level of NF-κB p65 was significantly elevated (39). These results suggest that the NF-κB signaling pathway may be a critical point connecting between PARP10 and ferroptosis. Therefore, we infected *Ct* for 24 hpi on the basis of ensuring transfection efficiency and analyzed NF-κB signaling pathway-related molecules by Western blot (Fig. 5a). The results showed that p65 and IκBα total protein did not change significantly. When PARP10 was knocked down, both p-p65/p65 and p-IκBα/IκBα ratios were increased and were more pronounced in Erastin-treated cells. GPX4 (glutathione peroxidase 4) has been shown to be the most critical regulator of ferroptosis and is the most critical in most cells to prevent accumulation of lipid hydroperoxide enzyme (40). Here, we found that knockdown of PARP10 resulted in activation of the NF-κB signaling pathway, which inhibited downstream GPX4 expression (Fig. 5b). To further elucidate the role of knocking down PARP10 in *Ct*-infected cells that resulted in the activation of the NF-κB signaling pathway, we observed the nuclear translocation phenomenon of NF-κB p65 by fluorescence microscopy. It was found that both knockdown of PARP10 and Erastin treatment induced nuclear translocation of p65 (Fig. 5c). In addition, to further confirm that the inhibition of PARP10’s ability to weaken the rescue of ferroptosis by *Ct* is directly related to the NF-κB signaling pathway, we examined the protein expression of GPX4 in the PARP10-interacted group using BAY 11-7082, an inhibitor of NF-κB. The results showed that the addition of BAY 11-7082 rescued the inhibition of GPX4 by siPARP10 (Fig. 5d). These results suggest that *Ct* inhibits ferroptosis by upregulating PARP10 to suppress the activation of the NF-κB signaling pathway.

## DISCUSSION

*Ct* is one of the most common bacterial pathogens of STIs and an important cause of infertility and poor pregnancy outcomes. The interaction of *Ct* with host cell death and survival pathways is an active area of research, and its fate during the course of an infection is closely linked to the viability and mode of death of the host cells in which it is present (41).

Iron is an essential trace element for the survival of all living organisms (42), but iron overload leads to intracellular oxidative stress and ferroptosis. Meeting its own iron requirements while preventing excessive iron harm is a key survival challenge for the intracellular bacteria *Ct*. In this study, we first confirmed the time effect of *Ct* in inhibiting ferroptosis, which is related to its growth and development cycle. The anti-ferroptosis property of *Ct* was demonstrated by its ability to maintain cell viability and inhibit the production of MDA, and this resistance was most evident at 24 hpi, which is similar to the anti-apoptotic property of *Ct* that has been demonstrated in the past (43, 44). In addition, iron overload is usually associated with oxidative stress, and this study found that *Ct* infection reduced intracellular Fe^2+^ levels. In fact, in *Ct*-infected cells, when the iron concentration is high enough, probably due to the high expression of the YtgABCD manipulator, which increases iron uptake, after obtaining Fe^2+^, *Ct* can transfer Fe²^+^ to Fe-S cluster biosynthesis or utilize iron in other metabolic processes (45). This may be an explanation for the ability of *Ct* to prevent Erastin-induced iron overload in HeLa cells. The central site of iron metabolism is known to be the mitochondria, and mitochondrial dysfunction leads to oxidative stress and cellular damage (30). We found that Erastin causes a decrease in mitochondrial membrane potential, mitochondrial wrinkling, and a reduction in mitochondrial cristae in cells, whereas *Ct* attenuated the mitochondrial damage caused by Erastin. Thus, *Ct* may disrupt cellular ferroptosis by remodeling host cell mitochondria and thereby disrupting cellular ferroptosis. In addition, Erastin inhibits *Ct* through separable morphological and functional effects. TEM directly showed that Erastin co-treatment reduces inclusion size and EB/RB ratio, indicating disrupted bacterial development. However, reinfection assays revealed that progeny harvested from Erastin-stressed cultures form normally sized inclusions in the absence of Erastin, demonstrating that the morphological suppression is reversible. Our findings suggest Erastin may cause a temporary developmental delay rather than irreversible blockage. Future TEM studies of progeny inclusions could determine whether ferroptosis specifically alters EB/RB maturation timing.

Up-regulation of PARP10 expression was detected in *Ct*-infected HeLa cells. The PARP family is a class of enzymes that play key roles in DNA repair, genome stability, and cell death. Clinically, PARP inhibitors can trigger ferroptosis, for example, Niraparib mediates ferroptosis and immune activation by enhancing c-GAS signaling in colorectal cancer models (46), and olaparib’s pharmacology promotes lipid peroxidation and ferroptosis with p53/SLC7A11 and thus is used in the treatment of wild-type ovarian cancer (47). In addition, PARP1 plays a role in oxidative stress and cell death (48), therefore, PARP10 may affect ferroptosis by modulating cellular redox status or metabolic pathways (49). Here, we found that lowering PARP10 increased susceptibility to ferroptosis. Knockdown of PARP10 increases cellular ROS production in addition to decreasing cell viability and increasing intracellular Fe^2+^. The increased ROS is most likely caused by the Fenton reaction induced by iron, which distinguishes it from other forms of cell death. ROS has both benefits and drawbacks for *Ct*, and respiratory bursts triggered by large or prolonged ROS production are toxic to pathogens. It has been shown that *Ct* induces ROS production within a few hours and then, after 9 h, can rapidly target NADPH oxidase activity to restore ROS to normal levels (50), and that 24 hpi host production of ROS contributes to *Ct* growth (51).

A close functional link between PARP10 and the NF-κB signaling pathway has been reported (35). Recent studies have indicated that activation of inflammation-related signaling pathways can lead to ferroptosis. Intervention of the NF-κB pathway has been demonstrated in various cellular and animal experiments to exert an anti-ferroptosis effect and attenuate the adverse effects of the disease (38). Earlier studies showed that, in contrast to *Chlamydia pneumoniae*, NF-κB activation was not observed in human epithelial cells infected with *Ct* (52, 53). These data provide a theoretical basis for us to explore whether PARP10 regulates the NF-κB pathway to exert ferroptosis resistance in the context of *Ct* infection. Knockdown of PARP10 in *Ct*-infected, Erastin-induced ferroptosis in HeLa cells revealed upregulation of phosphorylated IκBα as well as elevated levels of phosphorylated NF-κB p65, suggesting activation of the NF-κB signaling pathway. The released NF-κB dimers were translocated to the nucleus and regulated downstream GPX4 expression, however, whether NF-κB is a transcriptional regulator of GPX4 needs to be further verified. In addition, the use of BAY 11-7082, an inhibitor of NF-κB, reversed GPX4 levels to some extent, suggesting that PARP10 inhibition impaired the ability of *Ct* to rescue ferroptosis and was directly related to the NF-κB signaling pathway.

In conclusion, this study reveals for the first time a novel mechanism of *Ct*-regulated ferroptosis and extends the function of PARP10 from the traditional DNA repair and inflammation regulation to the field of pathogen infection, revealing its new role in host-pathogen interactions. However, the specific molecular mechanisms of how PARP10 is regulated by *Ct* to manipulate ferroptosis need to be further explored. By interfering with the PARP10/NF-κB signaling pathway or ferroptosis process, it may provide potential targets for the treatment of *Ct* infection. In addition, how *Ct* regulates ferroptosis, including the regulation of host iron metabolism, oxidative stress, and mitochondrial function, so as to shape a favorable environment for its own survival, is a worthy direction for future research.

## Data Availability

The data that support the findings of this study are available from the corresponding author upon request.

## SUPPLEMENTAL MATERIAL

Fig. S1 spectrum.03568-25-s0001.tiff Quantitative analysis of the mitochondrial red fluorescence to green fluorescence among groups. siPARP10 or siNC was transfected into HeLa cells, and the titer of infectious progeny was determined using an immunofluorescence assay with IFUs.

Supplemental material spectrum.03568-25-s0002.docx Fig. S1 legend.

ASM does not own the copyrights to Supplemental Material that may be linked to, or accessed through, an article. The authors have granted ASM a non-exclusive, world-wide license to publish the Supplemental Material files. Please contact the corresponding author directly for reuse.
