# Supplementary figures and images for: Chlamydia trachomatis inhibits NF-κB-dependent ferroptosis through PARP10 upregulation to promote reproduction
Source: Microbiol Spectr. 2026 May 21;14(7):e03568-25. doi: 10.1128/spectrum.03568-25 (PMC13340069; doi:10.1128/spectrum.03568-25)

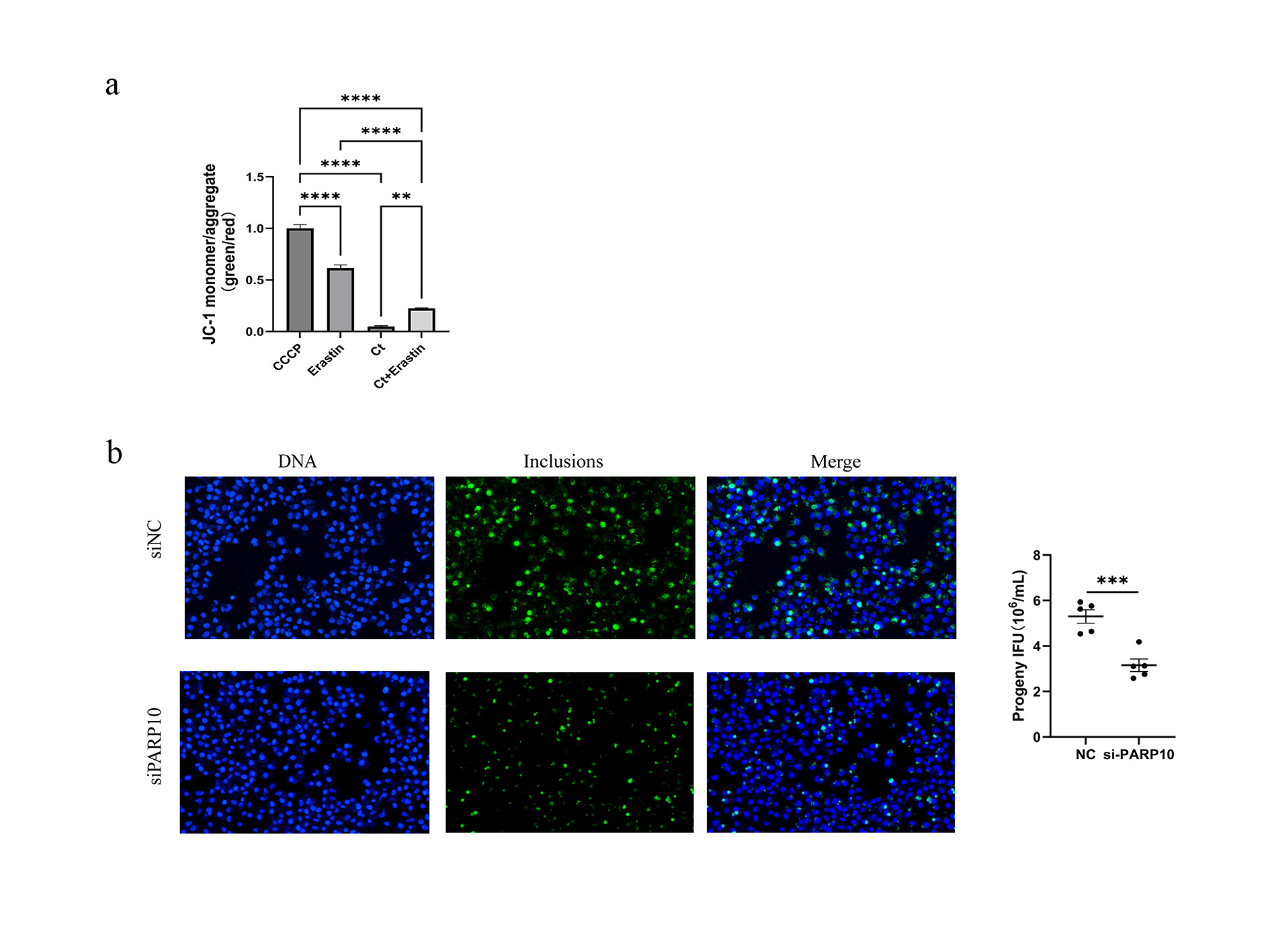

Supplement: Fig. S1 — Quantitative analysis of the mitochondrial red fluorescence to green fluorescence among groups. siPARP10 or siNC was transfected into HeLa cells, and the titer of infectious progeny was determined using an immunofluorescence assay with IFUs. [file spectrum.03568-25-s0001.tiff]
