# Supplementary material for: Chlamydia trachomatis inhibits NF-κB-dependent ferroptosis through PARP10 upregulation to promote reproduction
Source: Microbiol Spectr. 2026 May 21;14(7):e03568-25. doi: 10.1128/spectrum.03568-25 (PMC13340069; doi:10.1128/spectrum.03568-25)
Supplement: Supplemental material — Fig. S1 legend. [file spectrum.03568-25-s0002.docx]

**Figure S1**

**a.** Quantitative analysis of the mitochondrial red fluorescence to green fluorescence among groups. All values are denoted as means ± SEM from five independent photographs shot in each group. **b.** siRNA targeting PARP10 (siPARP10) or negative control siRNA (siNC) was transfected into HeLa cells, and the titer of infectious progeny was determined using an immunofluorescence assay with inclusion-forming units (IFUs). Data are expressed as mean ± SEM (n = 5) and analyzed by Student's t-test. **p* < 0.05; ***p* < 0.01; ****p* < 0.001;*****p* < 0.0001.
